# P018. No evidence of microstructural changes in patients with vestibular migraine: a diffusion tensor tract based spatial statistic (TBSS) study

**DOI:** 10.1186/1129-2377-16-S1-A161

**Published:** 2015-09-28

**Authors:** Antonio Russo, Laura Marcuccio, Francesca Conte, Giuseppina Caiazzo, Alfonso Giordano, Renata Conforti, Fabrizio Esposito, Gioacchino Tedeschi, Alessandro Tessitore

**Affiliations:** Department of Medical, Surgical, Neurological, Metabolic and Aging Sciences, Second University of Naples, Naples, Italy; MRI Research Center SUN-FISM, Second University of Naples, Naples, Italy; Institute for Diagnosis and Care “Hermitage Capodimonte”, Naples, Italy; Department of Medicine and Surgery, University of Salerno, Baronissi, SA Italy; Neuroradiology Unit, Department of Clinical and Experimental Medicine and Surgery, Second University of Naples, Naples, Italy

## Background

Vestibular migraine (VM) has been increasingly recognized as a possible cause of episodic vertigo [1], but its pathophysiology is still unclear. In our previous fMRI study, we had observed a significantly increased thalamic activation in patients with vestibular migraine (VM) during vestibular stimulation in comparison with patients with migraine without aura (MwoA) and healthy controls (HC) [2]. Recently, a voxel based morphometry (VBM) study has shown gray matter volume reduction in brain areas involved in pain and vestibular processing [3]. However, no studies have yet investigated white matter (WM) microstructural abnormalities in patients with VM.

## Objective

To investigate whole-brain and thalamic WM microstructural changes in patients with VM, compared with patients with MwoA and HC.

## Methods

By using magnetic resonance imaging and diffusion tensor imaging (DTI) with tract-based spatial statistic (TBSS) analysis [4], we analyzed WM integrity in twenty patients with VM, compared to twenty patients with MwoA and twenty HC. We performed a TBSS analysis generating fractional anisotropy (FA), mean diffusivity (MD) and radial diffusivity (RD) and axial diffusivity (AD) maps. TBSS was run with FA maps to create the “skeleton”, which represents the center of all fiber bundles in common to all subjects. The resulting statistical maps were thresholded at p < 0.05 corrected for multiple comparisons at a cluster level. Besides whole brain analyses, a region of interest (ROI) analysis was also performed to correlate the TBSS results with both thalamic standard anatomic ROI data and functional regions that were based on the results of our previous fMRI study.

## Results

Between-groups analyses did not reveal statistically significant differences in both whole-brain and bilateral thalamic ROI FA, MD, RD and AD values between patients with VM compared with patients with MwoA and HC (p < 0.05 corrected).

## Conclusions

Recent studies have demonstrated that the thalamus may play a major role in an abnormal information processing during ictal and interictal migraineous periods. Our previous fMRI study has clearly demonstrated an abnormal thalamic activation during vestibular processing in patients with VM. However, this functional phenomenon seems not be correlated to any structural connectivity changes since both whole-brain and thalamic ROI DTI analyses have not demonstrated significant differences between VM, MwoA and HC. Our preliminary data may support the hypothesis that thalamic functional changes may not be linked to, or alternatively, may precede structural abnormalities in patients with VM.

Written informed consent to publish was obtained from the patient(s).
